# Methyl *N*-(di­meth­oxy­phosphor­yl)carbamate

**DOI:** 10.1107/S1600536813017637

**Published:** 2013-06-29

**Authors:** Vladimir Ovchynnikov

**Affiliations:** a Department of Inorganic Chemistry, Kiev National Taras Shevchenko University, Vladimirskaya St. 64/13, Kiev 01601, Ukraine

## Abstract

In the title compound, CH_3_OC(O)NHP(O)(OCH_3_)_2_, the P atom has a slightly distorted tetra­hedral configuration. The mixed imide moiety can be described as *cisoid–transoid* in which the two opposing dipoles (P=O and C=O) are oriented with a O=C⋯P=O torsion angle of 150.88(18)°. In the crystal, molecules are linked by pairs of N—H⋯O hydrogen bonds, forming inversion dimers.

## Related literature

For the use of phospho­rylated carbamides as potential new ligands, see: Safin *et al.* (2009 ▶); Znovjyak *et al.* (2009 ▶); Sokolov *et al.* (2008 ▶). For their biological activity, see: Amirkhanov *et al.* (1996 ▶); Rebrova *et al.* (1984 ▶); Tsibulskaya & Orlacheva (1956 ▶). For P=O bond lengths, see: Mizrahi & Modro (1982 ▶): Amirkhanov *et al.* (1997 ▶). For the synthesis of the title compound, see: Kirsanov & Marenetc (1959 ▶). For short O⋯O contacts see: Bianchi *et al.* (2000 ▶); Zhurova *et al.* (2002 ▶)
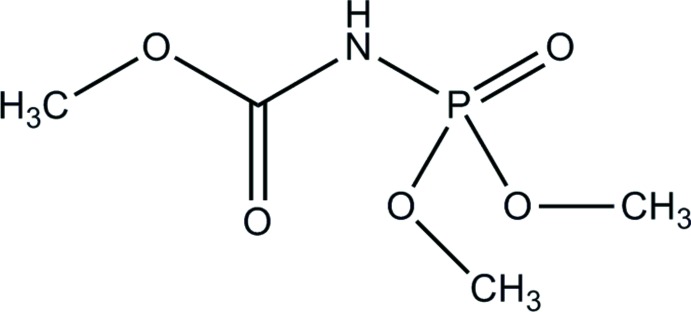


## Experimental

### 

#### Crystal data

C_4_H_10_NO_5_P
*M* *_r_* = 183.10
Triclinic, 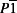

*a* = 6.441 (1) Å
*b* = 7.018 (1) Å
*c* = 9.298 (2) Å
α = 99.05 (3)°
β = 96.70 (3)°
γ = 100.54 (3)°
*V* = 403.46 (14) Å^3^
*Z* = 2
Mo *K*α radiation
μ = 0.32 mm^−1^
*T* = 293 K
0.30 × 0.30 × 0.25 mm

#### Data collection

Enraf–Nonius CAD-4 diffractometer
Absorption correction: ψ scan (North *et al.*, 1968 ▶) *T*_min_ = 0.910, *T*_max_ = 0.924
1417 measured reflections
1417 independent reflections
1322 reflections with *I* > 2σ(*I*)
3 standard reflections every 200 reflections intensity decay: 1%

#### Refinement

*R*[*F* ^2^ > 2σ(*F* ^2^)] = 0.048
*wR*(*F* ^2^) = 0.133
*S* = 1.04
1417 reflections
101 parameters
H-atom parameters constrained
Δρ_max_ = 0.74 e Å^−3^
Δρ_min_ = −0.37 e Å^−3^

### 

Data collection: *CAD-4 EXPRESS* (Enraf–Nonius, 1994 ▶); cell refinement: *CAD-4 EXPRESS*; data reduction: *XCAD4* (Harms & Wocadlo, 1996 ▶); program(s) used to solve structure: *SHELXS97* (Sheldrick, 2008 ▶); program(s) used to refine structure: *SHELXL97* (Sheldrick, 2008 ▶); molecular graphics: *ORTEP-3* (Farrugia, 2012 ▶); software used to prepare material for publication: *WinGX* (Farrugia, 2012 ▶).

## Supplementary Material

Crystal structure: contains datablock(s) I, global. DOI: 10.1107/S1600536813017637/gg2115sup1.cif

Structure factors: contains datablock(s) I. DOI: 10.1107/S1600536813017637/gg2115Isup2.hkl

Supplementary material file. DOI: 10.1107/S1600536813017637/gg2115Isup3.cml

Additional supplementary materials:  crystallographic information; 3D view; checkCIF report

## Figures and Tables

**Table:** 

P1—O1	1.451 (2)
P1—O4	1.556 (2)
P1—O3	1.573 (2)
P1—O2	3.126 (2)
P1—N1	1.658 (2)
O2—O4	2.938 (3)

**Table:** 

O1—P1—O4	117.63 (12)
O1—P1—O3	109.28 (13)
O4—P1—O3	101.56 (12)
O1—P1—N1	109.55 (12)
O4—P1—N1	108.84 (12)
O3—P1—N1	109.57 (13)

**Table 2:** Hydrogen-bond geometry (Å, °)

*D*—H⋯*A*	*D*—H	H⋯*A*	*D*⋯*A*	*D*—H⋯*A*
N1—H1*N*⋯O1^i^	0.86	1.99	2.847 (3)	171

Symmetry code: (i) 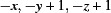.

**Fig. 1.**
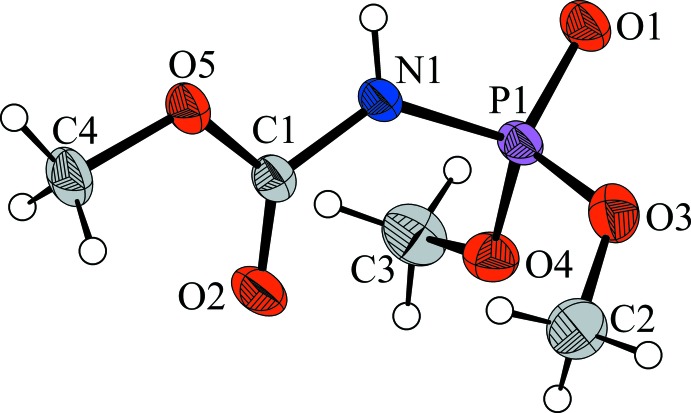
A view of the title compound showing the atom-numbering scheme and displacement ellipsoids drawn at the 30% probability level.

**Fig. 2.**
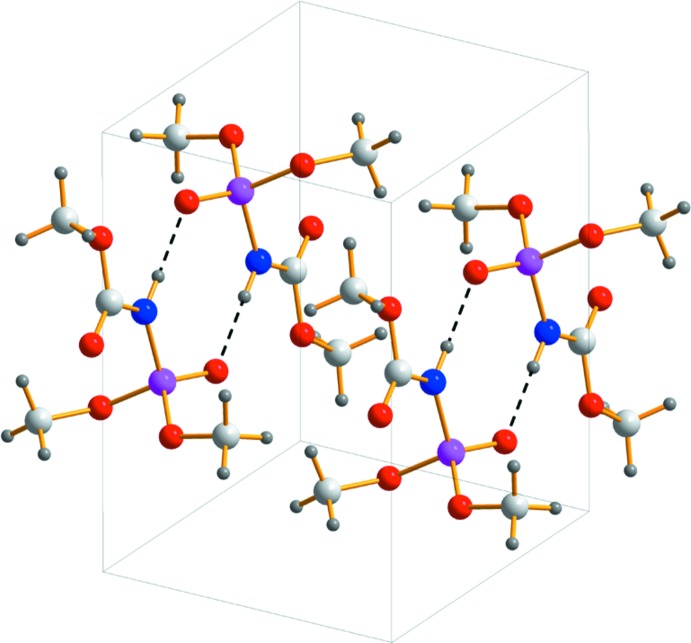
Intermolecular contacts, hydrogen bonds shown as dashed lines.

## supplementary crystallographic information

### Comment

Phosphorylated carbamate of the general formula ROC(O)NHP(O)*R*_2_ are potential new ligands for metal ions (Sokolov *et al.* 2008). Many of these compounds also show biological activity (Amirkhanov *et al.* 1996, Rebrova *et al.* 1984, Tsibulskaya *et al.* 1956). This work reports the structure of methyl(dimethoxyphosphoryl)carbamate (I) (C_4_H_10_NO_5_P).

In the title compound (I), the phosphorus atom has a slightly distorted tetrahedral configuration. The average values of the angles OPN and OPO in the molecule are close to the tetrahedral, with the exception O3—P1—O4 and O1–P1–O4, which can be explained by interaction of nucleophilic carbonyl oxygen atom O2 with electrophilic phosphorus atom P1, corresponding distance less than the sum of the Van der Waals Radii 3.3 Å. There is repulsion between the oxygen atoms O2 and O4 distorting the tetrahedral environment of the phosphorus atom, the O···O distance is less than the sum of the Van der Waals radii 3.04 Å. Short O···O interactions have also been reported for dinitramide anion (Zhurova *et al.*, 2002) and dimanganese decacarbonyl (Bianchi *et al.*, 2000).

The P1–O1 and P1–N1 bond lengths for compound (I) have values 1.451 Å and 1.658 Å, which are typical for carbacylamidophosphates with ether-type substituents (Amirkhanov *et al.* 1997). The mixed imide moiety can be described as *cisoid-transoid* in which the two opposing dipoles (P=O and C=O) are oriented with torsion angle O2=C1···P1=O1 150.88 (18)° (Fig.1).

Molecules are linked in centrosymmetric dimmers by hydrogen bonds of the phosphoryl oxygen atoms and the hydrogen atoms of the C(O)N(H)P(O) groups of neighboring molecules (Fig.2, Table 2).

Fragment C4 O5 C1 O2 N1 P1 is practically planar, with deviations from the mean plane not exceeding 0.052 (2) Å. The O1 and O4 atoms are adjacent to it with deviations of 0.453 (3) Å and 0.582 (3) Å, respectively. The P=O bond has an angle of deviation from this plane of 23.4 (2)°.

### Experimental

All chemicals were commercial products of reagent grade, used without further purification. Methyl(dimethoxyphosphoryl)carbamate (I) was prepared as in (Kirsanov *et al.* 1959). Single crystals of (I) were prepared by slow crystallization from benzene solution.

### Refinement

The H atoms bonded to C and N were located in differnce Fourier maps but subsequently introduced in calculated positions and treated as riding on their parent atoms (C or N) with C–H = 0.98 Å with *U*_iso_(H) = 1.5 and N–H = 0.86 Å with *U*_iso_ (H) = 1.2 *U*_eq_.

### Crystal data

**Table:**

C_4_H_10_NO_5_P	*Z* = 2
*M**_r_* = 183.10	*F*(000) = 192
Triclinic, *P*1	*D*_x_ = 1.507 Mg m^−^^3^
Hall symbol: -P 1	Melting point: 337 K
*a* = 6.441 (1) Å	Mo *K*α radiation, λ = 0.71069 Å
*b* = 7.018 (1) Å	Cell parameters from 1635 reflections
*c* = 9.298 (2) Å	θ = 2.2–25.0°
α = 99.05 (3)°	µ = 0.32 mm^−^^1^
β = 96.70 (3)°	*T* = 293 K
γ = 100.54 (3)°	Block, colourless
*V* = 403.46 (14) Å^3^	0.30 × 0.30 × 0.25 mm

### Data collection

**Table:**

Enraf–Nonius CAD-4 diffractometer	1322 reflections with *I* > 2σ(*I*)
Radiation source: fine-focus sealed tube	*R*_int_ = 0.000
Graphite monochromator	θ_max_ = 25.5°, θ_min_ = 2.2°
ω/Θ scans	*h* = 0→7
Absorption correction: ψ scan (North *et al.*, 1968)	*k* = −8→8
*T*_min_ = 0.910, *T*_max_ = 0.924	*l* = −10→10
1417 measured reflections	3 standard reflections every 200 reflections
1417 independent reflections	intensity decay: 1%

### Refinement

**Table:**

Refinement on *F*^2^	Secondary atom site location: difference Fourier map
Least-squares matrix: full	Hydrogen site location: inferred from neighbouring sites
*R*[*F*^2^ > 2σ(*F*^2^)] = 0.048	H-atom parameters constrained
*wR*(*F*^2^) = 0.133	*w* = 1/[σ^2^(*F*_o_^2^) + (0.0766*P*)^2^ + 0.3565*P*] where *P* = (*F*_o_^2^ + 2*F*_c_^2^)/3
*S* = 1.04	(Δ/σ)_max_ = 0.001
1417 reflections	Δρ_max_ = 0.74 e Å^−^^3^
101 parameters	Δρ_min_ = −0.37 e Å^−^^3^
0 restraints	Extinction correction: *SHELXL97* (Sheldrick, 2008), Fc^*^=kFc[1+0.001xFc^2^λ^3^/sin(2θ)]^-1/4^
Primary atom site location: structure-invariant direct methods	Extinction coefficient: 0.38 (3)

### Special details

**Table:**

Geometry. All s.u.'s (except the s.u. in the dihedral angle between two l.s. planes) are estimated using the full covariance matrix. The cell s.u.'s are taken into account individually in the estimation of s.u.'s in distances, angles and torsion angles; correlations between s.u.'s in cell parameters are only used when they are defined by crystal symmetry. An approximate (isotropic) treatment of cell s.u.'s is used for estimating s.u.'s involving l.s. planes.
Refinement. Refinement of *F*^2^ against ALL reflections. The weighted *R*-factor *wR* and goodness of fit *S* are based on *F*^2^, conventional *R*-factors *R* are based on *F*, with *F* set to zero for negative *F*^2^. The threshold expression of *F*^2^ > 2σ(*F*^2^) is used only for calculating *R*-factors(gt) *etc*. and is not relevant to the choice of reflections for refinement. *R*-factors based on *F*^2^ are statistically about twice as large as those based on *F*, and *R*- factors based on ALL data will be even larger.

### Fractional atomic coordinates and isotropic or equivalent isotropic displacement parameters (Å^2^)

**Table:**

	*x*	*y*	*z*	*U*_iso_*/*U*_eq_	
P1	0.12950 (10)	0.49103 (10)	0.73507 (7)	0.0377 (3)	
O1	−0.0897 (3)	0.4420 (3)	0.6611 (2)	0.0544 (6)	
O4	0.1726 (3)	0.5989 (3)	0.8985 (2)	0.0502 (6)	
O3	0.2109 (4)	0.2955 (3)	0.7485 (3)	0.0576 (6)	
O2	0.5735 (3)	0.7814 (3)	0.8088 (2)	0.0574 (6)	
O5	0.5569 (3)	0.8030 (3)	0.5700 (2)	0.0503 (6)	
N1	0.2820 (3)	0.6223 (3)	0.6387 (3)	0.0420 (6)	
H1N	0.2292	0.6164	0.5484	0.050*	
C3	0.1084 (6)	0.7823 (5)	0.9409 (4)	0.0648 (9)	
H3A	0.1479	0.8264	1.0453	0.097*	
H3B	−0.0436	0.7643	0.9155	0.097*	
H3C	0.1780	0.8790	0.8903	0.097*	
C2	0.4229 (6)	0.2930 (5)	0.8115 (5)	0.0668 (9)	
H2A	0.4385	0.1591	0.8085	0.100*	
H2B	0.4501	0.3612	0.9119	0.100*	
H2C	0.5229	0.3572	0.7566	0.100*	
C1	0.4822 (4)	0.7395 (4)	0.6847 (3)	0.0388 (6)	
C4	0.7664 (5)	0.9297 (5)	0.6001 (4)	0.0560 (8)	
H4A	0.8054	0.9672	0.5105	0.084*	
H4B	0.8684	0.8605	0.6400	0.084*	
H4C	0.7647	1.0455	0.6700	0.084*	

### Atomic displacement parameters (Å^2^)

**Table:**

	*U*^11^	*U*^22^	*U*^33^	*U*^12^	*U*^13^	*U*^23^
P1	0.0299 (4)	0.0452 (5)	0.0342 (5)	0.0002 (3)	0.0036 (3)	0.0054 (3)
O1	0.0403 (11)	0.0678 (13)	0.0464 (13)	−0.0103 (9)	0.0021 (9)	0.0123 (10)
O4	0.0469 (11)	0.0594 (12)	0.0423 (13)	0.0087 (9)	0.0053 (9)	0.0069 (9)
O3	0.0582 (13)	0.0456 (11)	0.0650 (15)	0.0035 (9)	0.0062 (11)	0.0084 (10)
O2	0.0387 (11)	0.0747 (15)	0.0468 (14)	−0.0092 (10)	−0.0027 (9)	0.0066 (10)
O5	0.0385 (11)	0.0550 (12)	0.0500 (13)	−0.0104 (8)	0.0027 (8)	0.0138 (9)
N1	0.0351 (11)	0.0490 (12)	0.0355 (13)	−0.0065 (9)	−0.0007 (9)	0.0100 (10)
C3	0.063 (2)	0.0623 (19)	0.064 (2)	0.0168 (16)	0.0072 (16)	−0.0071 (16)
C2	0.065 (2)	0.0559 (18)	0.081 (3)	0.0213 (16)	0.0028 (18)	0.0142 (17)
C1	0.0321 (12)	0.0388 (13)	0.0426 (16)	0.0031 (10)	0.0034 (11)	0.0054 (11)
C4	0.0374 (15)	0.0576 (17)	0.066 (2)	−0.0091 (12)	0.0085 (13)	0.0117 (15)

### Geometric parameters (Å, º)

**Table:**

P1—O1	1.451 (2)	N1—C1	1.377 (3)
P1—O4	1.556 (2)	N1—H1N	0.8600
P1—O3	1.573 (2)	C3—H3A	0.9600
P1—N1	1.658 (2)	C3—H3B	0.9600
P1—O2	3.126 (2)	C3—H3C	0.9600
O4—C3	1.434 (4)	C2—H2A	0.9600
O4—O2	2.938 (3)	C2—H2B	0.9600
O3—C2	1.427 (4)	C2—H2C	0.9600
O2—C1	1.198 (3)	C4—H4A	0.9600
O5—C1	1.326 (3)	C4—H4B	0.9600
O5—C4	1.444 (3)	C4—H4C	0.9600
			
O1—P1—O4	117.63 (12)	O4—C3—H3B	109.5
O1—P1—O3	109.28 (13)	H3A—C3—H3B	109.5
O4—P1—O3	101.56 (12)	O4—C3—H3C	109.5
O1—P1—N1	109.55 (12)	H3A—C3—H3C	109.5
O4—P1—N1	108.84 (12)	H3B—C3—H3C	109.5
O3—P1—N1	109.57 (13)	O3—C2—H2A	109.5
O1—P1—O2	149.38 (10)	O3—C2—H2B	109.5
O4—P1—O2	68.54 (9)	H2A—C2—H2B	109.5
O3—P1—O2	97.78 (10)	O3—C2—H2C	109.5
N1—P1—O2	45.52 (9)	H2A—C2—H2C	109.5
C3—O4—P1	121.2 (2)	H2B—C2—H2C	109.5
C3—O4—O2	94.46 (19)	O2—C1—O5	125.1 (2)
P1—O4—O2	81.92 (10)	O2—C1—N1	125.7 (3)
C2—O3—P1	123.2 (2)	O5—C1—N1	109.1 (2)
C1—O2—O4	87.22 (16)	O5—C4—H4A	109.5
C1—O2—P1	60.15 (15)	O5—C4—H4B	109.5
C1—O5—C4	116.0 (2)	H4A—C4—H4B	109.5
C1—N1—P1	128.4 (2)	O5—C4—H4C	109.5
C1—N1—H1N	115.8	H4A—C4—H4C	109.5
P1—N1—H1N	115.8	H4B—C4—H4C	109.5
O4—C3—H3A	109.5		
			
O1—P1—O4—C3	−56.6 (3)	N1—P1—O2—C1	−3.4 (2)
O3—P1—O4—C3	−175.8 (2)	O1—P1—O2—O4	108.3 (2)
N1—P1—O4—C3	68.7 (2)	O3—P1—O2—O4	−99.42 (13)
O2—P1—O4—C3	90.3 (2)	N1—P1—O2—O4	150.72 (16)
O1—P1—O4—O2	−146.92 (12)	O1—P1—N1—C1	161.9 (2)
O3—P1—O4—O2	93.90 (11)	O4—P1—N1—C1	32.0 (3)
N1—P1—O4—O2	−21.63 (11)	O3—P1—N1—C1	−78.2 (3)
O1—P1—O3—C2	177.8 (2)	O2—P1—N1—C1	3.27 (19)
O4—P1—O3—C2	−57.2 (3)	O4—O2—C1—O5	169.5 (3)
N1—P1—O3—C2	57.8 (3)	P1—O2—C1—O5	−178.1 (3)
O2—P1—O3—C2	12.3 (3)	O4—O2—C1—N1	−8.8 (3)
C3—O4—O2—C1	−98.7 (2)	P1—O2—C1—N1	3.6 (2)
P1—O4—O2—C1	22.27 (18)	C4—O5—C1—O2	1.1 (4)
C3—O4—O2—P1	−120.9 (2)	C4—O5—C1—N1	179.7 (2)
O1—P1—O2—C1	−45.8 (3)	P1—N1—C1—O2	−7.5 (4)
O4—P1—O2—C1	−154.1 (2)	P1—N1—C1—O5	173.93 (19)
O3—P1—O2—C1	106.5 (2)		

### Hydrogen-bond geometry (Å, º)

**Table:**

*D*—H···*A*	*D*—H	H···*A*	*D*···*A*	*D*—H···*A*
N1—H1*N*···O1^i^	0.86	1.99	2.847 (3)	171

Symmetry code: (i) −*x*, −*y*+1, −*z*+1.
